# Prophylactic ALA Administration Attenuates Liver and Lung Injury in CLP-Induced Sepsis in Mice Through CSE/H_2_S Dependent Pathways

**DOI:** 10.3390/ijms27209341

**Published:** 2026-10-21

**Authors:** Aqsa Shahid, Amy Scott-Thomas, Stephen Chambers, Andrew Miller, Madhav Bhatia

**Affiliations:** Department of Pathology and Molecular Medicine, University of Otago, Christchurch 8140, New Zealand; shaaq042@student.otago.ac.nz (A.S.); amy.scott-thomas@otago.ac.nz (A.S.-T.); steve.chambers@otago.ac.nz (S.C.);

**Keywords:** cystathionine γ-lyase, alpha lipoic acid, cecal ligation and puncture, hydrogen sulfide, anti-inflammatory agent

## Abstract

Sepsis is a life-threatening syndrome characterized by a dysregulated host immune response to infection. Sepsis development usually results in organ dysfunction and multiple organ failure, including the liver and lungs. Alpha-lipoic acid (ALA) is a well-known anti-inflammatory agent. However, its effects on the endogenous cystathionine γ-lyase/hydrogen sulfide (CSE/H_2_S) pathway during cecal ligation and puncture (CLP)-induced sepsis remain unclear. In this study, the protective effects of ALA in wild-type (WT) and CSE knockout (CSE^−/−^) mice were investigated. Mice were randomly divided into six experimental groups, including (*a*) WT sham (*b*) CSE^−/−^ sham (*c*) WT CLP (*d*) CSE^−/−^ CLP (*e*) WT CLP + ALA.P and (*f*) CSE^−/−^ CLP + ALA.P (*n* = 8 in each group). ALA (100 mg/kg) was administered intraperitoneally one hour before CLP surgery. Eight hours after CLP induction, mice were euthanized, and blood, liver and lung tissues were collected. Body weight changes, proinflammatory cytokine and chemokine concentrations (including tumor necrosis factor-alpha (TNF-α), monocyte chemoattractant protein-1 (MCP-1) and macrophage inflammatory protein-2 alpha (MIP-2α), myeloperoxidase (MPO) activity, histopathological changes, plasma H_2_S concentrations, and CSE expression in the liver and lung were measured. Severe liver and lung damage was characterized by markedly increased levels of MCP-1, MIP-2 α, TNF-α, and myeloperoxidase (MPO) activity, compared to WT sham control and CSE^−/−^ groups (*p* < 0.0001). WT CLP-induced septic mice showed liver and lung injury as evidenced by hepatocyte swelling and ballooning degeneration with liver sinusoidal congestion, as well as pulmonary intra-alveolar and interstitial edema associated with thickening of alveolar septa and leucocyte infiltration, as compared to WT sham control and CSE^−/−^ groups (*p* < 0.001). The WT-CLP group also showed a significant increase in hepatic and pulmonary expression of the H_2_S-producing enzyme (CSE) with elevated levels of plasma H_2_S concentrations, as compared to the WT sham group (*p* < 0.0001). Prophylactic ALA administration markedly improved these changes in the WT group. ALA also significantly decreased plasma H_2_S concentrations and hepatic and pulmonary CSE expression as compared to the WT CLP group (*p* < 0.01). However, ALA administration did not confer additional significant protective effects in CSE^−/−^ mice, which supports the hypothesis that the effects of ALA are mediated through the modulation of the CSE/H_2_S pathway. In conclusion, this is the first experimental evidence showing that ALA prevents sepsis-associated organ injury at least partly by the downregulation of hepatic and pulmonary cystathionine γ-lyase expression.

## 1. Introduction

Sepsis is characterized by a dysregulated host response to infection, and any infectious stimulus can trigger an uncontrolled immune response. This causes an excessive production of chemokines, cytokines, oxidative stress markers, and neutrophil infiltration [1]. Activation of immune cells, neutrophil infiltration and excessive production of pro-inflammatory mediators significantly contribute to tissue damage in vital organs such as the liver and lungs [2,3].

Cecal ligation and puncture (CLP) is the most commonly used rodent model to induce sepsis because it closely mimics the pathophysiology of human sepsis [4,5,6,7,8]. Cecum perforation results in the continuous leakage of intestinal bacteria into the peritoneal cavity, leading to polymicrobial peritonitis, a systemic inflammatory response, and ultimately sepsis. Furthermore, this CLP model reflects the early hyperdynamic cardiovascular changes and systemic release of pro-inflammatory mediators, as observed in human sepsis [9].

Hydrogen sulfide (H_2_S) is a gasotransmitter; other gases playing this role are nitric oxide (NO) and carbon monoxide (CO). H_2_S, a major contributor to several physiological and pathological effects, is predominantly endogenously synthesized by cystathionine γ-lyase (CSE). H_2_S is recognized as an important signaling molecule due to its ability to act as both a pro-inflammatory and anti-inflammatory mediator during several diseases. It has been reported that H_2_S significantly inhibited fibroblast pyroptosis and activation of the NLRP3 inflammasome and facilitated wound healing in a streptozotocin-induced diabetic rat model [10]. A growing body of evidence suggests that H_2_S exerts pro-inflammatory effects in several inflammatory conditions, including CLP-induced sepsis in mice and sepsis in human patients [4,11,12]. These studies have shown that CLP-induced sepsis increased the expression of CSE, which in turn exacerbates the inflammatory response by the upregulation of pro-inflammatory cytokines (TNF-α and IL-6) and chemokines (MCP-1 and MIP-2α) in the liver and lungs. To investigate the functions of H_2_S, different approaches to inhibit H_2_S synthesis have been used. These include pharmacological agents, propargylglycine (PAG), H_2_S-producing gene silencing through siRNA, and CSE gene deletion [13,14,15]. Interestingly, inhibition of H_2_S production by PAG, CSE gene silencing, and gene deletion decrease levels of pro-inflammatory chemokines and cytokines in the liver and lungs. Collectively, these findings suggest that the CSE/H_2_S pathway plays a pro-inflammatory role in sepsis and opens a new horizon for developing potential therapeutic agents that target H_2_S synthesis.

Alpha-lipoic acid (ALA), a potent organosulfur compound, has well-documented anti-inflammatory and antioxidant activities [16]. ALA is considered an ideal antioxidant due to its unique amphiphilic nature and confers protective effects in both oxidized (ALA) and reduced form (dihydrolipoic acid, DHLA) [17]. Studies have shown the therapeutic potential of ALA in inflammatory conditions, including sepsis, acute pancreatitis, rheumatoid arthritis, and asthma [18,19,20,21]. Several clinical trials have also highlighted the protective effects of ALA therapy. ALA administration significantly reduced the serum levels of TNF-α, IL-6 and IL-1β by downregulating Toll-like receptor 4 (TLR-4) and NF-κB expression in osteoarthritis patients compared to controls [22]. However, the underlying mechanisms, particularly the effect of ALA on endogenous H_2_S production in sepsis, have not been completely elucidated. Recently, we have demonstrated that ALA suppressed the production of pro-inflammatory mediators and modulated CSE expression in LPS-stimulated RAW 264.7 macrophages [23]. These results also made a case for investigating the effect of ALA administration in an animal model of sepsis in order to determine the effects on the complex pathophysiology and associated damage to different organs.

In this paper, we have investigated the effect of administration of ALA on hepatic and pulmonary inflammation in CLP-induced sepsis in mice. We have also investigated whether the effects of ALA are mediated through modulation of the CSE/H_2_S pathway.

## 2. Results

### 2.1. Effect of Prophylactic ALA Administration on Body Weight in Mice Following CLP-Induced Sepsis

**Figure 1:**
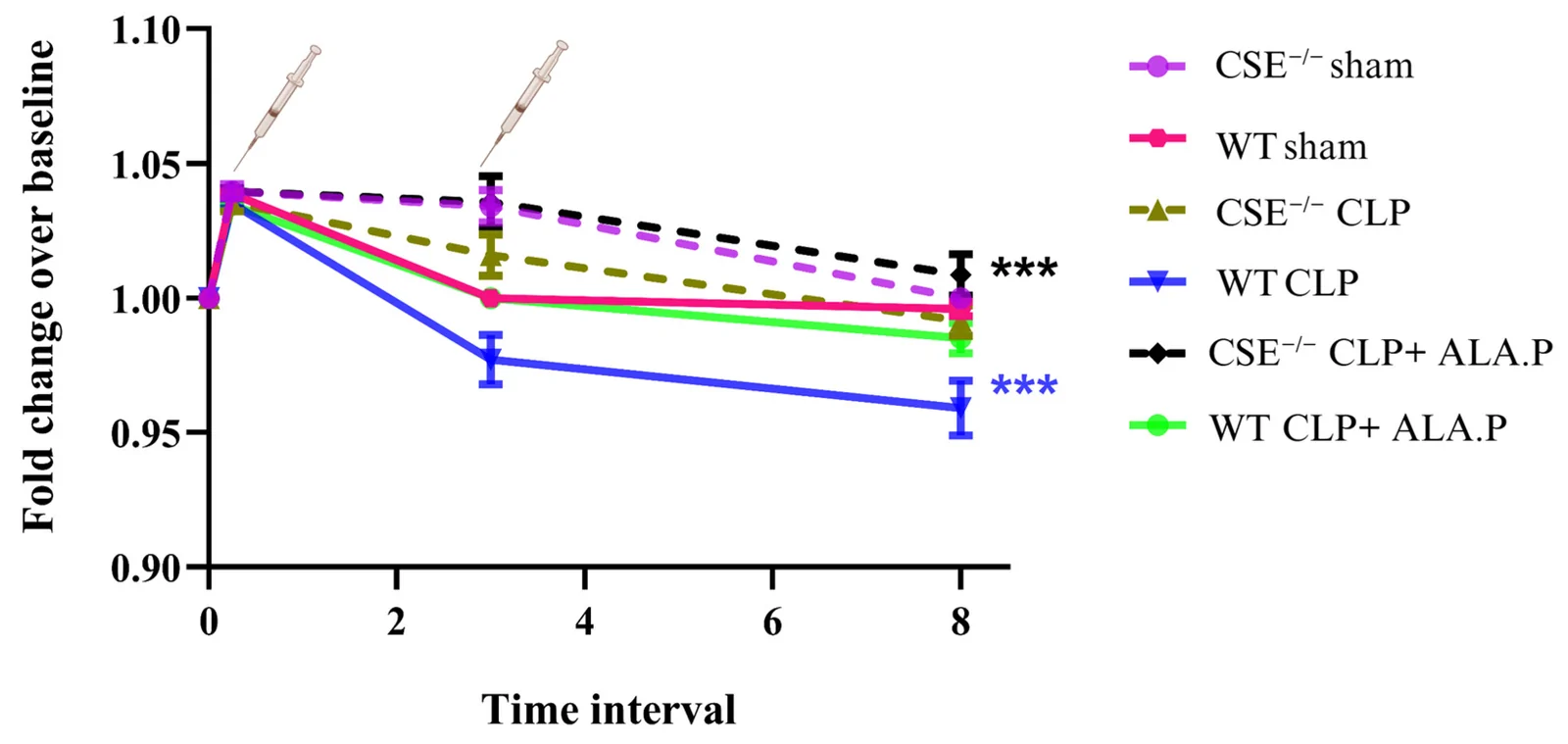
Mean Body weight changes in WT and CSE^−/−^ mice. Changes in the mice’s body weight were documented up to 8 h after CLP. The WT sham and CSE^−/−^ sham groups did not show any significant changes in body weight. Whereas a progressive decline in body weight was observed in the WT CLP group. The groups receiving intraperitoneal administration of ALA (100 mg/kg) before CLP did not show any significant change in weight. In contrast, there was a significant increase in weight in ALA-treated CSE^−/−^ mice. Fluctuations in weight at 15 min and 3 h are due to the administration of normal saline immediately after surgery—Temgesic and normal saline, respectively. Data are presented as mean ± SEM (*n* = 8 mice per group). A linear mixed model was used to evaluate the differences between groups. Statistical significance was determined at *** *p* < 0.001 and non-significant (ns).

The mean body weight did not change significantly over time in the WT sham group. A significant decrease in weight was observed in the WT CLP group (*p* < 0.001). Prophylactic administration of ALA did not significantly affect weight in the WT CLP + ALA.P group as compared to the WT CLP group alone. No significant weight loss was observed in the CSE^−/−^ sham group. A small but non-significant trend towards change was observed in the CSE^−/−^ CLP group. However, prophylactic ALA administration prevented weight loss in CSE^−/−^ CLP + ALA.P (*p* = 0.002) as compared to the CSE^−/−^ CLP group, suggesting that the protective effect of ALA on weight loss could be independent of CSE (Figure 1).

### 2.2. Effect of Prophylactic ALA Administration on MPO Activity in the Liver and Lungs in Mice Following CLP-Induced Sepsis

**Figure 2:**
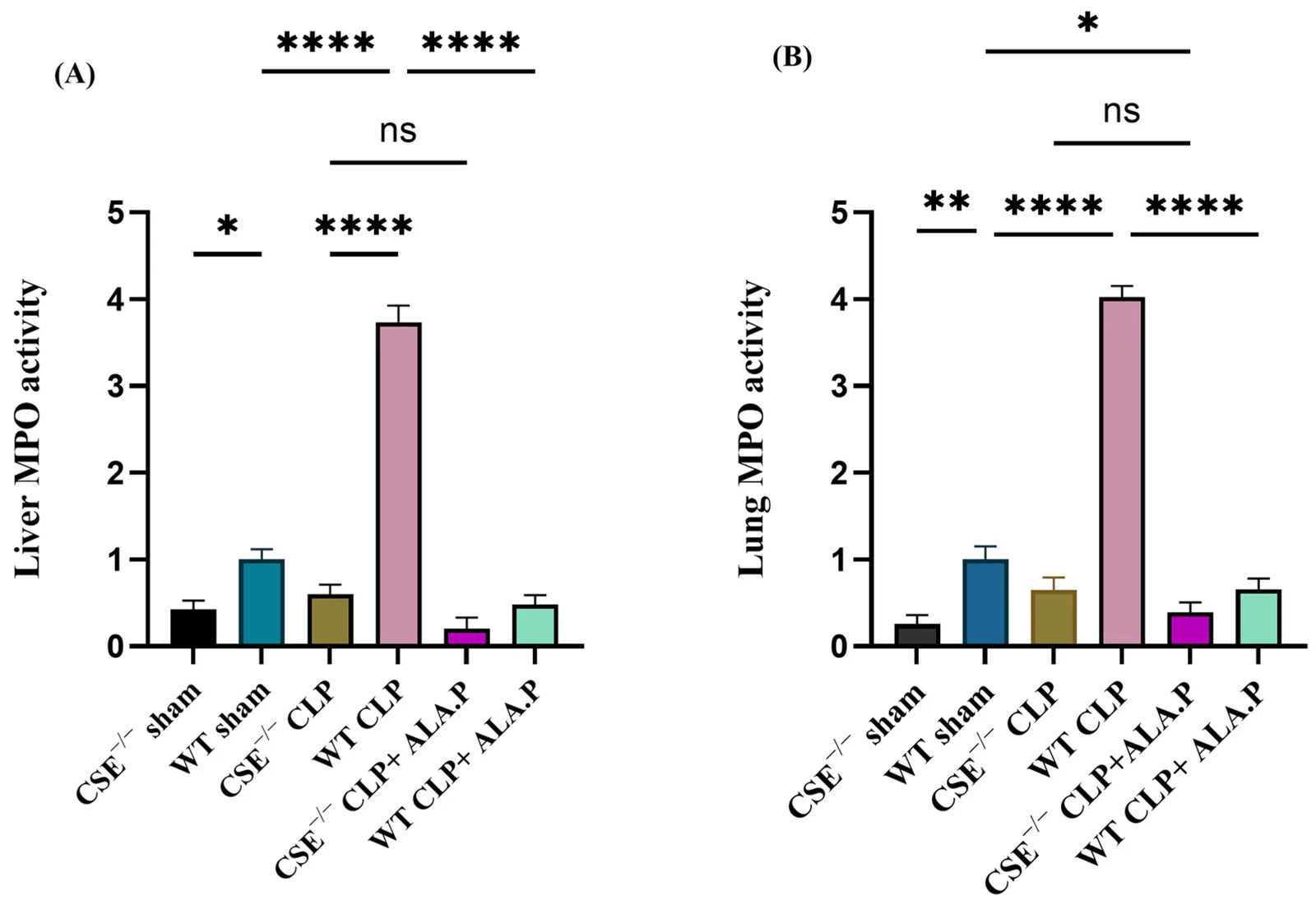
Effect of prophylactic ALA administration on liver and lung MPO activity after 8 h of CLP-induced sepsis in WT and CSE^−/−^ mice (**A**,**B**). MPO activity is expressed as a fold increase over the control (sham) group. CLP induction markedly increased MPO activity in the liver and lungs in the WT group. This increase in MPO activity was significantly reduced in CSE^−/−^ groups and by prophylactic administration of ALA in the WT group. Data are presented as mean ± SEM (*n* = 8 mice per group). One-way ANOVA with post hoc Tukey’s test was used to evaluate differences among all groups. Statistical significance was determined at * *p* < 0.05; ** *p* < 0.01; **** *p* < 0.0001; and non-significant (ns).

A significant increase in MPO activity was observed in both organs in the WT CLP group compared to the WT sham and CSE^−/−^ groups. Prophylactic administration of ALA in the WT group significantly attenuated this increase in MPO activity in both organs. A small but non-significant numerical trend towards improvement was observed in CSE^−/−^ mice after ALA administration as compared to CSE^−/−^ CLP group (Figure 2).

### 2.3. Effect of Prophylactic ALA Administration on the Levels of Pro-Inflammatory Cytokine TNF-α and Chemokines MCP-1 and MIP-2α in the Liver and Lungs of Mice Following CLP-Induced Sepsis

**Figure 3:**
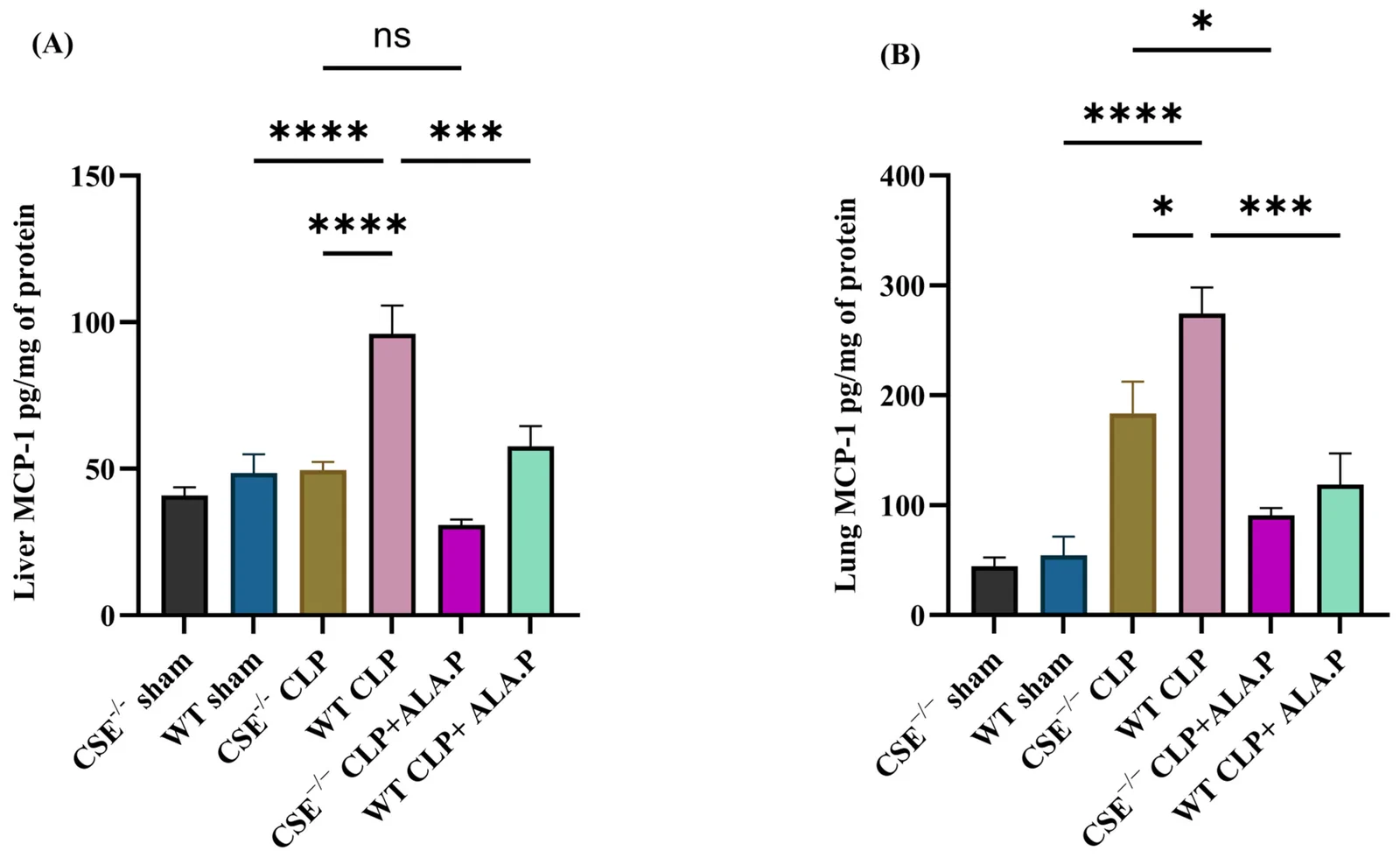

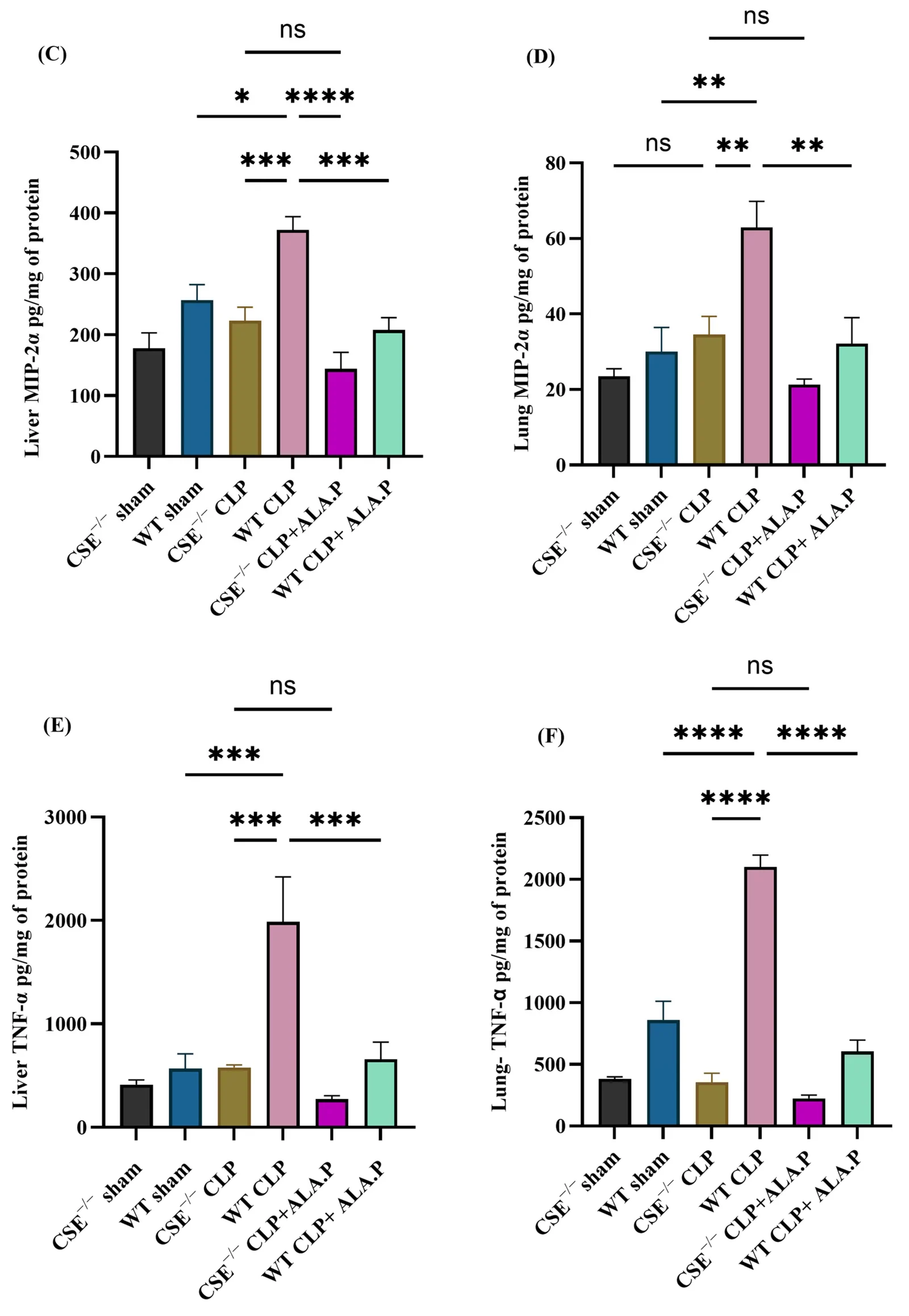
Effect of prophylactic ALA administration on chemokines (MCP-1 and MIP-2α) and cytokine (TNF-α) concentrations after 8 h of CLP-induced sepsis in WT and CSE^−/−^ mice in liver and lungs (**A**–**F**). CLP induction significantly elevated MCP-1, MIP-2α and TNF-α concentrations in the liver and lungs in the WT group compared to the WT sham and CSE^−/−^ groups. ALA administration before surgery significantly reduced the increase in cytokine and chemokine concentrations in the WT CLP group. A small but non-significant difference was observed in the CSE^−/−^ group after ALA administration. Data are presented as mean ± SEM (*n* = 8 mice per group). One-way ANOVA with a post hoc Tukey’s test was used to evaluate differences among all groups. Statistical significance was determined at * *p* < 0.05; ** *p* < 0.01; *** *p* < 0.001; **** *p* < 0.0001; and non-significant (ns).

Proinflammatory chemokines (MCP-1 and MIP-2 α) and cytokine (TNF-α) were significantly increased in both the liver and lungs of the WT CLP mice group compared to WT sham and CSE^−/−^ groups. Prophylactic administration of ALA significantly decreased the elevated concentrations of MCP-1, MIP-2α and TNF-α in both organs in WT mice. A small but non-significant (except MIP-2α) numerical trend towards improvement was observed in CSE^−/−^ mice after ALA administration as compared to the CSE^−/−^ CLP group Figure 3).

### 2.4. Effect of Prophylactic ALA Administration on Sepsis-Associated Organ Injury in CLP-Induced Septic WT and CSE^−/−^ Mice—Histological Evidence

**Figure 4:**
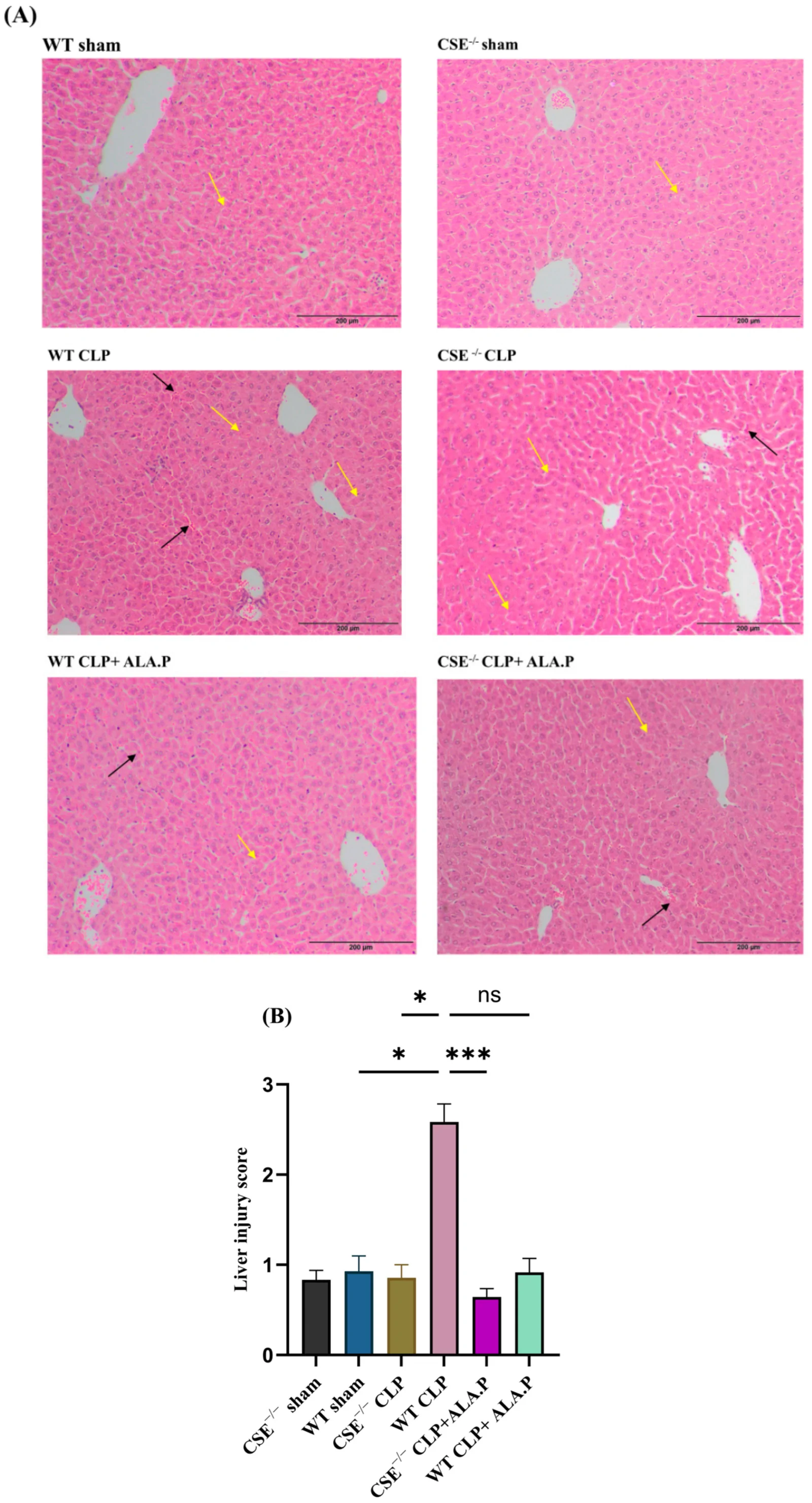
Effect of prophylactic ALA administration on liver injury after 8 h of CLP-induced sepsis in WT and CSE^−/−^ mice (**A**,**B**). Representative histopathological images of liver samples obtained after H & E staining showed sinusoidal congestion (black arrows), ballooning degeneration, and hepatocyte swelling (yellow arrows) in the WT sepsis group as compared to WT sham, CSE^−/−^ sham and CLP group. ALA administration significantly reduced these changes in the WT CLP group. A small but non-significant difference was observed in the CSE^−/−^ group after ALA administration. The Kruskal–Wallis test followed by Dunn’s multiple comparisons test was used for non-parametric data. All data are represented as mean ± SEM (*n* = 6 mice per group). Statistical significance was determined at * *p* < 0.05; *** *p* < 0.001; and non-significant (ns).

**Figure 5:**
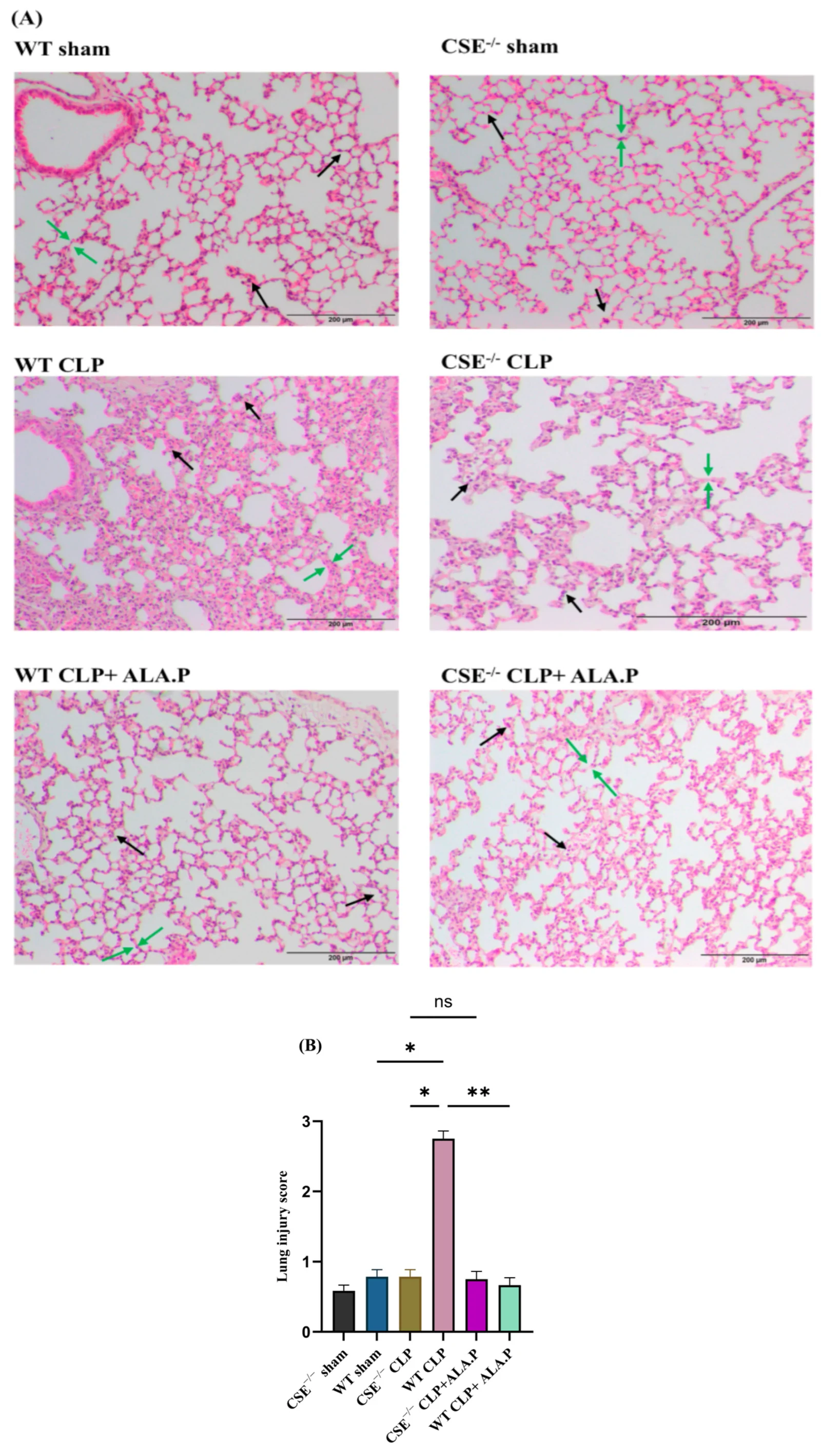
Effect of prophylactic ALA administration on lung injury after 8 h of CLP-induced sepsis in WT and CSE^−/−^ mice (**A**,**B**). Representative histopathological images of lung samples obtained after H & E staining showed diffuse alveolar damage (DAD), including intra-alveolar edema and interstitial edema with thickening of alveolar septa (green arrows), and neutrophil infiltration (black arrows), in the WT CLP group as compared to the WT sham and CSE^−/−^ groups. ALA administration significantly reduced these changes in the WT CLP group. No significant differences were observed in the CSE^−/−^ mice group after ALA administration. The Kruskal–Wallis test followed by Dunn’s multiple comparisons test was used for non-parametric data. All data are represented as mean ± SEM (*n* = 6 mice per group). Statistical significance was determined at * *p* < 0.05; ** *p* < 0.01; and non-significant (ns).

Histological changes in the liver and lung tissue samples were observed after H&E staining. Sepsis-associated liver injury, evidenced by hepatocyte swelling and ballooning degeneration, focal lobular necrosis and sinusoidal congestion, was observed in samples from WT mice with CLP-induced sepsis. Mice from WT sham, CSE^−/−^ sham, and CSE^−/−^ CLP groups had significantly less liver injury. Prophylactic ALA administration in WT CLP showed significantly fewer changes in liver histology and prevented all the changes caused by sepsis. A small but non-significant numerical trend towards improvement was observed in CSE^−/−^ mice after ALA administration as compared to the CSE^−/−^ CLP group (Figure 4A,B). Lung histology showed features of diffuse alveolar damage (DAD), including intra-alveolar edema and interstitial edema with thickening of alveolar septa, interstitial and intra-alveolar infiltration of neutrophils and focal intra-alveolar fibrin deposition, in the WT CLP group compared to the WT sham, CSE^−/−^ sham, and CSE^−/−^ CLP. Prophylactic treatment with ALA significantly attenuated these sepsis-associated changes caused in the WT CLP group. Furthermore, no significant differences were observed in the CSE^−/−^ CLP group with ALA administration (Figure 5A,B).

### 2.5. Effect of Prophylactic ALA Administration on CSE Expression in CLP-Induced Sepsis-Associated Organ Injury

**Figure 6:**
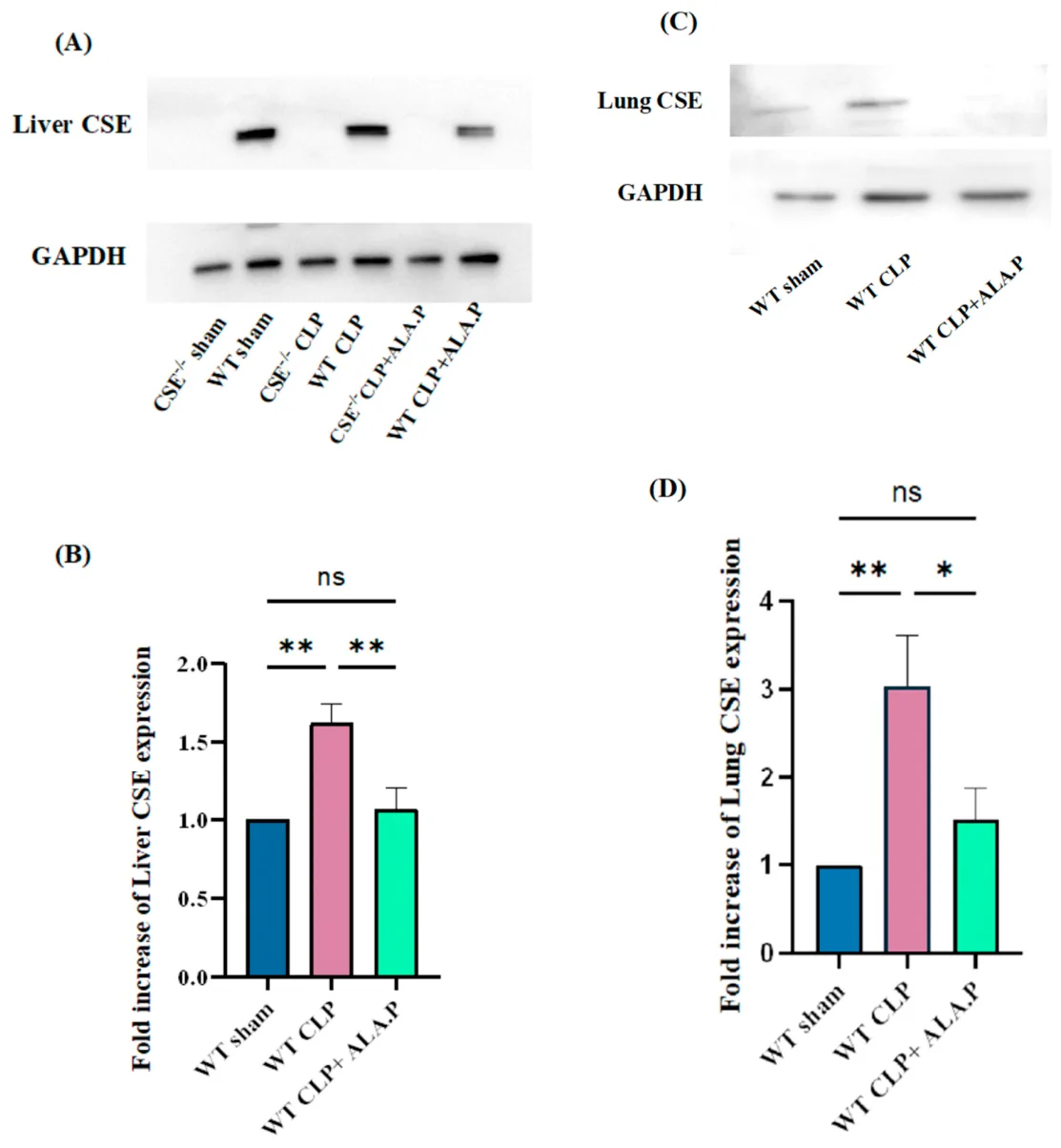
The effects of ALA on CSE expression in the liver (**A**,**B**) and lung (**C**,**D**) after CLP-induced sepsis. CSE expression is expressed as a fold increase over the sham group. CSE expression was significantly higher in the liver and lungs following CLP-induced sepsis, whereas ALA administration significantly decreased this elevated expression in both organs (**A**–**D**). CSE band intensity was normalized against GAPDH (**A**,**C**). Representative Western blot images were obtained from six independent experiments. All the data are represented as mean ± SEM (*n* = 6 mice per group). A one-way ANOVA with a post hoc Tukey’s test was used. Statistical significance was determined at * *p* < 0.05; ** *p* < 0.01; and non-significant (ns).

CSE expression was significantly increased in WT CLP-induced septic mice liver (Figure 6A,B) and lung (Figure 6C,D) compared to the sham group. Prophylactic ALA administration significantly suppressed hepatic and pulmonary CSE expression in WT CLP-induced septic mice. Glyceraldehyde-3-phosphate dehydrogenase (GAPDH) was used as a loading control to compare CSE expression (Figure 6A,C).

### 2.6. Effect of Prophylactic ALA Administration on Plasma H_2_S Concentrations in CLP-Induced Sepsis

**Figure 7:**
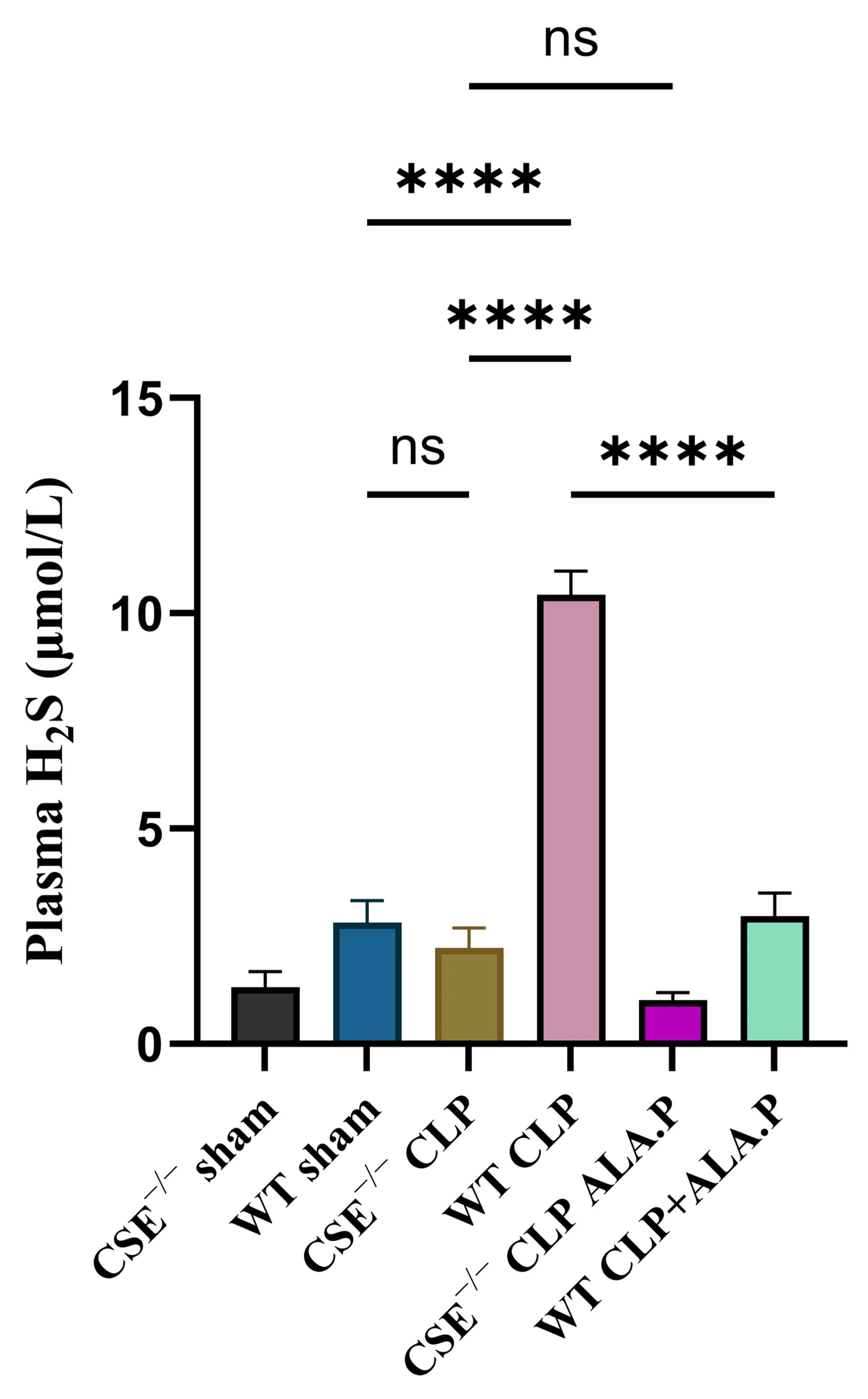
Mouse plasma H_2_S concentrations. A significant increase in H_2_S concentration was observed in the WT CLP group as compared to the WT sham and CSE^−/−^ groups. ALA administration significantly decreased the H_2_S concentration in WT CLP mice. All the data are represented as mean ± SEM (*n* = 5 mice per group). A one-way ANOVA with a post hoc Tukey’s test was used. Statistical significance was determined at **** *p* < 0.0001 and non-significant (ns).

The plasma H_2_S concentrations were significantly increased in WT mice following CLP-induced sepsis compared with WT sham and CSE^−/−^ mice groups. Prophylactic ALA administration significantly reduced the increased plasma H_2_S concentration in WT mice (Figure 7).

## 3. Discussion

Despite advances in critical care, sepsis and associated multiple organ dysfunction remain major causes of morbidity and mortality worldwide. Sepsis-associated acute lung injury (ALI) is among the most common complications and is associated with poor clinical outcomes. The present study demonstrates that prophylactic administration of ALA attenuated CLP-induced liver and lung injury in WT mice. ALA administration did not significantly influence weight loss following CLP-induced sepsis in WT mice but prevented weight loss in CSE^−/−^ mice. ALA reduced inflammatory mediator production, decreased MPO activity, preserved tissue architecture, and reduced plasma H_2_S levels and CSE expression in WT mice. These findings suggest that modulation of CSE/H_2_S contributes to the protective effects of ALA.

This study has shown that administration of ALA one hour before CLP surgery significantly reduced the sepsis-associated liver and lung injury in WT and CSE^−/−^ mice. ALA administration did not significantly influence sepsis-associated weight loss following CLP-induced sepsis in the WT mice. Whereas ALA administration prevented such weight loss in CSE^−/−^ mice. This finding highlights that CSE gene deletion and prophylactic ALA administration were associated with improved weight outcomes compared to WT mice. On the other hand, in WT mice, ALA administration alone was insufficient to prevent CLP-induced sepsis-associated weight loss at the eight-hour time point.

Induction of sepsis in WT mice significantly increased the production of proinflammatory mediators (TNF-α, MCP-1, MIP-2α). In the present study, CLP-induced sepsis significantly increased hepatic and pulmonary levels of TNF-α, MCP-1, and MIP-2α in WT mice, whereas deletion of the CSE gene markedly attenuated these responses. These findings support previous evidence implicating H_2_S as an important mediator of sepsis-associated organ injury. Notably, prophylactic administration of ALA significantly reduced the production of these pro-inflammatory mediators in WT mice. ALA administration did not significantly affect the production of these mediators in CSE^−/−^ mice. These findings are consistent with our previous work demonstrating that ALA suppresses MCP-1, IL-6, and TNF-α release in LPS-stimulated RAW 264.7 macrophages [23]. Similarly, our data are consistent with the previous findings indicating that ALA alleviates liver and lung injury following CLP-induced sepsis through suppression of inflammatory mediator production [20,24].

It is well documented that neutrophil activation and tissue infiltration are hallmarks of sepsis and play an important role in the development of tissue injury, particularly in acute lung injury (ALI). Myeloperoxidase (MPO) is widely used as a marker to measure neutrophil activation and infiltration during inflammatory diseases [25]. Our study has shown that MPO levels in the liver and lungs were markedly elevated in WT mice compared to CSE^−/−^ mice following CLP-induced sepsis. These findings highlight the contribution of the CSE/H_2_S pathway in sepsis. Prophylactic administration of ALA significantly decreased the hepatic and pulmonary MPO activity in WT mice. However, no additional statistically significant differences were observed in CSE^−/−^ mice after ALA administration. Our findings are consistent with previous reports showing that ALA administration attenuated the increase in MPO activity in the liver, lungs and kidneys in the CLP-induced sepsis model [26].

H_2_S is known as a major contributor to the pathology of CLP-induced sepsis and associated multiple organ failure [27,28]. In this study, we have shown that plasma H_2_S levels and hepatic and pulmonary CSE expression were significantly upregulated in WT mice following CLP-induced sepsis. Prophylactic administration of ALA markedly reduced plasma H_2_S levels and downregulated hepatic and pulmonary CSE expression, suggesting that suppression of the CSE/H_2_S pathway contributes to its anti-inflammatory effects. These findings are consistent with our earlier findings indicating that anti-inflammatory effects of ALA are associated with the modulation of the CSE/H_2_S pathway [23].

H_2_S is recognized as an important biomarker due to its pleiotropic nature. Controlled release of H_2_S shows anti-inflammatory effects by inhibiting the synthesis of endogenous H_2_S. On the other hand, rapid release of H_2_S is associated with the exacerbation of inflammation. In vivo and in vitro models, exogenous and endogenous H_2_S significantly promoted angiogenesis, wound healing and improved cardiac function [29,30]. The anti-inflammatory effects of H_2_S in a rat model of colitis were observed. Suppression of colonic H_2_S resulted in the aggravation of colitis [31]. A significant downregulation of cyclooxygenase-2 mRNA and prostaglandin synthesis, with increased mucosal damage in the colon, was observed in healthy rats after inhibiting H_2_S synthesis. On the other hand, intracolonic H_2_S administration markedly decreased colonic mRNA TNF-α expression and disease severity [31]. In one study, exogenous administration of sodium hydrosulfide (an H_2_S donor) improved survival rate, cognitive impairment, upregulated heme oxygenase-1 expression and reduced neuronal apoptosis in the CLP-induced septic mice model. These findings suggest that a controlled dose of H_2_S exhibits neuroprotective effects [32]. A substantial body of evidence suggests that H_2_S contributes to the progression of inflammatory diseases, including sepsis. Pharmacological inhibition of CSE by propargylglycine (PAG-CSE inhibitor) or genetic deletion of CSE significantly suppressed the production of cytokines, chemokines, adhesion molecules, and MPO activity in liver and lungs following CLP-induced sepsis [14]. Moreover, in RAW 264.7 macrophages stimulated with LPS, an increase in the activation of extracellular signal-regulated kinase 1/2 (ERK1/2) and the transcription factor nuclear factor-*κ*B (NF-*κ*B) pathway was observed. Notably, silencing of the CSE gene by small interfering RNA (siRNA) has shown the downregulation of these pathways [13]. In studies that investigated the translational potential or the role of H_2_S in human sepsis, it has been shown that plasma H_2_S levels are significantly increased in patients with sepsis [5,11]. Collectively, these findings show that H_2_S is an important pro-inflammatory regulator in the pathogenesis of sepsis. Previous studies have shown that the therapeutic effects of ALA are also dependent on the modulation of H_2_S-mediated pathways. In the type 2 diabetes mellitus rat model, ALA administration has been shown to increase hepatic sulfane sulfur levels, H_2_S formation and upregulation of CSE expression. This increase in hepatic H_2_S levels was associated with mitigation of diabetes-induced hepatic injury [33]. ALA administration conferred anti-inflammatory effects through sulfane sulfur metabolism, as evidenced by improved vascular permeability and neutrophil infiltration in a zymosan-induced peritonitis mouse model [34]. Recently, it was shown that ALA treatment significantly inhibited CSE expression in RAW 264.7 macrophages before and after LPS stimulation at different time points [23]. Furthermore, in carrageenan-induced paw edema, ALA administration decreased paw edema in mice. On the other hand, co-treatment with glibenclamide (an anti-inflammatory drug that blocks K_ATP_ channels, involved in H_2_S signaling) inhibited the effects of ALA and exacerbated the paw edema [35].

Protective effects of ALA are associated with several interconnected signaling pathways including NF-*κ*B, nuclear factor erythroid 2-related factor 2 (Nrf2)/antioxidant response element (ARE), p38 and ERK [20,36]. The results of the present study provide strong evidence that prophylactic ALA administration can attenuate the increase in plasma H_2_S levels and CSE expression in liver and lungs in WT mice following CLP-induced sepsis. These findings suggest that the protective effects of ALA are mediated, at least in part, through H_2_S pathways. Despite these promising findings, several limitations should be acknowledged. Firstly, ALA was administered prophylactically before CLP induction; further studies are needed to elucidate the effects of ALA after the onset of sepsis.

Secondly, animals were monitored for eight hours only, which is sufficient to observe early inflammatory responses. Future studies are needed to investigate survival outcomes and organ protection. Thirdly, ALA was administered as a single dose, and an eight-hour time point was selected to assess all outcomes. Future studies with repeated doses and later time points are needed to clarify the systemic effects. Furthermore, additional downstream pathways responsible for the protective effects of ALA during sepsis should be investigated. Despite these limitations, the present study points to the potential of ALA as a therapeutic agent for sepsis and shows that its protective effect is mediated, at least in part, via the CSE/H_2_S pathway. This research opens up new avenues for the treatment of sepsis, a major health problem worldwide.

## 4. Materials and Methods

### 4.1. Animals

All animal experiments in the present study were conducted in compliance with the ethical guidelines set by the University of Otago Animal Welfare Office and Ethics Committee under the approved protocol (animal use protocol number AUP-24-17). Two mouse strains, including wild type (WT) (C57BL/6J) and a CSE^−/−^ knockout (C57/Bl6 background), were used within this study. The WT mice were ordered through the Integrated Animal Research Management System (IARMS) from Biomedical Research Facilities (BRF) Dunedin. These mice arrived at the Christchurch Animal Research Area (CARA) aged 5 weeks to ensure proper acclimatization before commencing experiments. The CSE ^−/−^ mice were generously gifted by Dr Isao Ishii from Showa Pharmaceutical University, Tokyo [37]. All animals used in this study were adult male mice between 8 and 12 weeks. The colony was maintained at CARA, and mice were ordered through IARMS as per protocol. Mice were randomly divided into six experimental groups, including (i) WT sham (ii) CSE^−/−^ sham (iii) WT CLP (iv) CSE^−/−^ CLP (v) WT CLP + ALA.P and (vi) CSE^−/−^ CLP + ALA.P. Each experimental group consisted of 8 mice (*n* = 8).

### 4.2. Sepsis Induction

Cecal ligation and puncture (CLP) method with a few modifications to an already established protocol was used in this study [14]. Each mouse was administered buprenorphine (Temgesic Sapphire Bioscience, Redfern, Australia) at 0.2 mg/kg subcutaneously 45 min prior to general anesthesia. Mice were anesthetized with 5% isoflurane and 55 psi O_2_ flowing at 1 L/min, which was maintained at 1–2% isoflurane during surgery. A 5% chlorhexidine solution was applied to the abdomen after the removal of fur by shaving. A small midline incision in the abdomen was made through the skin and then through the Linea alba. The cecum was exposed carefully and ligated with DemeSilk thread at the end. The cecum was perforated at two different sites using a 22-gauge needle. Following perforation, a small amount of fecal content from each hole was extruded, and the bowel was carefully repositioned. Local anesthetic, i.e., lignocaine (4 mg/kg), was administered by dripping into the abdomen before closing the peritoneum. The peritoneum and skin were sutured separately with 5.0 DemeSilk sutures. A stock concentration of ALA was prepared in 96% ethanol and serially diluted twice using normal saline for a final concentration of 100 mg/kg. ALA solutions were prepared fresh and used immediately to prevent degradation. ALA was administered intraperitoneally one hour before CLP surgery in both WT CLP + ALA.P and CSE^−/−^ CLP + ALA.P groups. Sham mice underwent the same surgical protocol except for bowel perforation. Weight changes were monitored carefully for the entire 15 min post-surgery and after every hour till euthanizing the animals. 1 mL saline was injected subcutaneously immediately after surgery to prevent dehydration. All animals were given buprenorphine (Temgesic) at 0.2 mg/kg and 1 mL normal saline (1 mL) subcutaneously 3 h post-surgery. After eight hours, pentobarbital (150 mg/kg) was administered intraperitoneally to euthanize the mice.

### 4.3. Tissue Collection

After euthanasia, blood samples were carefully aspirated from the right ventricle using heparinized syringes to prevent clotting. Plasma was separated by centrifugation at 10,000× *g* for 10 min at 4 °C and stored at −80 °C for further analysis. Liver and lung tissues were divided into two portions immediately following collection. One portion was fixed in 4% buffered paraformaldehyde for 24 h, followed by dehydration through a series of graded ethanol concentrations and paraffin embedding for histological analysis. The remaining portion was stored at −80 °C for subsequent quantitative analysis.

### 4.4. Myeloperoxidase (MPO) Activity

To determine the leukocyte infiltration in the liver and lung tissues, MPO activity was measured. The tissue pellet was resuspended in 50 mM phosphate buffer supplemented with 0.5% *w/v* hexadecyltrimethylammonium bromide (Sigma-Aldrich, Dallas, TX, USA) to facilitate membrane disruption. These suspensions then underwent three freeze-thaw cycles and were subsequently sonicated at 20% power and 80% pulse for 40 s. After sonication, the samples were centrifuged at 20,000× *g* for 15 min at 4 °C. A 50 μL supernatant aliquot was carefully transferred to a 96-well plate, and 100 μL of tetramethylbenzidine (TMB) was added. The plate was incubated at room temperature for two minutes, then 50 μL of 2 M H_2_SO_4_ was added to each well to stop the reaction. Absorbance was measured at 450 nm and 570 nm using a microplate spectrophotometer. Protein content of each sample was determined using the Bradford assay (Thermo Fisher Scientific Waltham, MA, USA). Results of MPO activity are expressed as fold increase relative to the control group.

### 4.5. Enzyme-Linked Immunosorbent Assay

Liver and lung tissues were homogenized by adding approximately 50 mg of tissue to chilled 20 mM sodium phosphate buffer (pH 7.4). Sterilized scissors were used to cut the tissue into smaller segments, and then it was further homogenized by a tissue homogenizer for approximately one minute. The tissue was then centrifuged at 10,000× *g* for 10 min at 4 °C. To investigate the levels of TNF-α, MCP-1 and MIP-2α, a sandwich ELISA was performed. The levels of these pro-inflammatory mediators were measured using specific DuoSet ELISA Kits (R&D System Minneapolis, Minneapolis, MN, USA) according to the manufacturer’s instructions. The concentrations of these mediators were corrected for the total protein content by Bradford protein assay.

### 4.6. Histopathology

For histological analysis, 3 µm tissue sections were prepared using a Leica rotary microtome (HistoCore BIOCUT R, Leica Biosystems, Mt Waverley, Australia) and stained with hematoxylin/eosin (H&E). To evaluate liver and lung injury, slides were scored in a double-blind manner with the assistance of an anatomical pathologist (A.M.). To assess liver injury, several pathological hallmarks, including hepatocyte swelling and ballooning degeneration, sinusoidal congestion, and focal hepatocyte necrosis, were assessed in four random fields in each slide at 10× magnification (*n* = 6) [38]. To assess lung injury, American Thoracic Society guidelines were used [39]. Pathological hallmarks of diffuse alveolar damage (DAD), including intra-alveolar and interstitial edema with alveolar thickening and neutrophil infiltration, were assessed in four random fields in each slide at 10× magnification (*n* = 6).

### 4.7. Western Blotting

Liver and lung tissues were homogenized. Briefly, approximately 50 mg of liver and lung tissues were homogenized in chilled radioimmunoprecipitation assay (RIPA) buffer supplemented with protease inhibitors. Tissues were centrifuged at 10,000× *g* for 10 min at 4 °C. Clear supernatant was used to check protein expression. Western blotting was performed as previously described [23] using primary antibodies CTH against (CAT—1:1000; mouse monoclonal antibody; Abnova Taipei, Taiwan, cat. no. H00001491-M01) and GAPDH (1:10,000; rabbit polyclonal antibody; ThermoFisher catalog no PA1-987). The corresponding secondary antibodies (Goat-anti-mouse HRP and Goat-anti-rabbit HRP; Santa Cruz, TX, USA) were used.

### 4.8. Plasma H_2_S Concentrations

Plasma H_2_S concentrations were measured according to the manufacturer’s instructions (ThermoFisher Scientific, Waltham, MA, USA). Briefly, 100 µL of buffer solution was added to the sample and control tubes. A 100 µL aliquot of serum and 100 µL of Milli-Q water were added to the sample and control tubes, respectively. Then, 100 µL of alkali reagent was added to both tubes and centrifuged at 12,000× *g* at 4 °C for ten minutes. 150 µL of Milli-Q water was added to all tubes and again centrifuged at 12,000× *g* at 4 °C for ten minutes. After centrifuging, 100 µL of buffer solution and chromogenic agent were added to all tubes and mixed for ten seconds. Amounts of 20 µL of ferric salt reagent and 100 µL of protein precipitator were added to all tubes and mixed for three seconds. The solution was centrifuged at 12,000× *g* at 4 °C for ten minutes, and 250 µL of supernatant was added to the corresponding wells. This solution was kept at room temperature for ten minutes, and the absorbance was measured at 665 nm.

### 4.9. Statistical Analysis

All data presented in this study are reported as mean ± standard error of the mean (SEM). To compare the differences between various groups, a one-way ANOVA followed by post hoc Tukey’s test was performed. If the data were not normally distributed, the non-parametric Kruskal–Wallis test was used. GraphPad Prism software (version 10.6.1 (892), San Diego, CA, USA) was used to perform statistical analysis in this study. Statistical significance was defined as * *p* < 0.05; ** *p* < 0.01; *** *p* < 0.001; **** *p* < 0.0001; and non-significant (ns).

## 5. Conclusions

**Figure 8:**
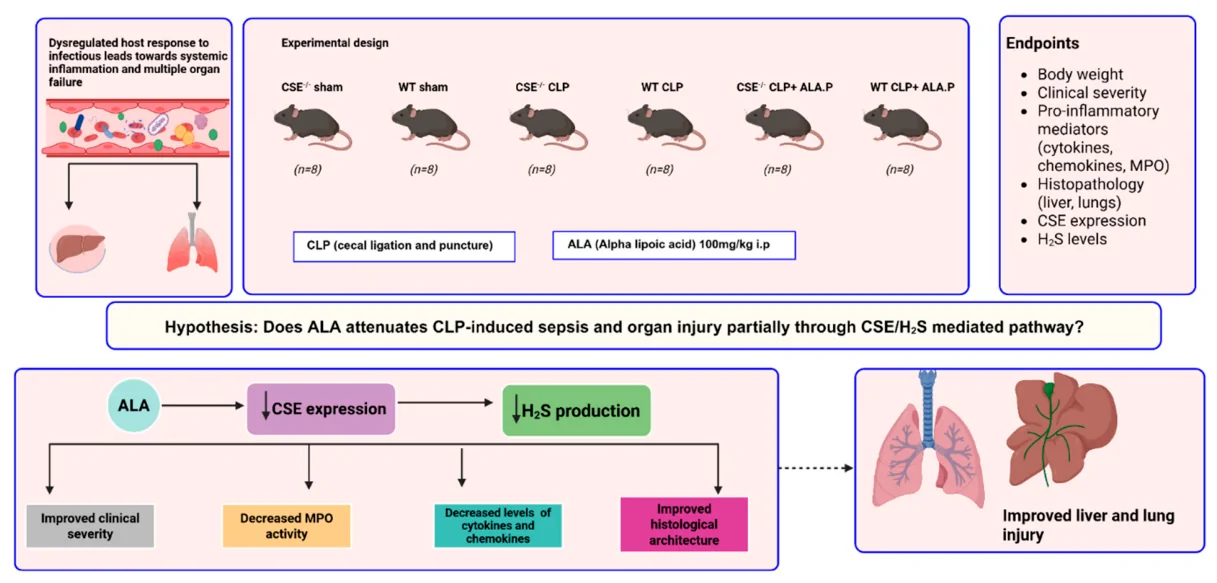
CLP-induced sepsis showed significant damage to the liver and lungs. ALA administration significantly ameliorated organ injury as evidenced by decreased cytokine and chemokine levels, MPO activity, and improved histological architecture. ALA administration also reduced the plasma H_2_S concentrations and CSE expression in liver and lungs.

In conclusion, the results presented in this paper show that the CSE/H_2_S pathway plays a key role in the progression of CLP-induced sepsis. There is a consistent pattern demonstrating that prophylactic ALA administration can significantly attenuate liver and lung injury in CLP-induced sepsis. These effects are associated with downregulation of CSE expression in WT mice and with CSE-independent mechanisms in CSE^−/−^ mice (Figure 8). As H_2_S acts as an important proinflammatory regulator in sepsis, treatments such as ALA that target both H_2_S-dependent inflammation together with other pathways offer a treatment strategy to protect against the devastating effects of sepsis.

## Data Availability

All data generated or analyzed during this study are included in this published article.
